# ASO Author Reflections: Sentinel Lymph Node Biopsy Trend in Melanoma: The More the Merrier

**DOI:** 10.1245/s10434-019-07660-w

**Published:** 2019-09-05

**Authors:** Mary-Ann El Sharouni, Arjen Witkamp, Vigfús Sigurdsson, Paul J. van Diest

**Affiliations:** 1 Department of Dermatology, University Medical Centre Utrecht, Utrecht University, Utrecht, The Netherlands; 2 Department of Surgery, University Medical Centre Utrecht, Utrecht University, Utrecht, The Netherlands; 3 Department of Pathology, University Medical Centre Utrecht, Utrecht University, Utrecht, The Netherlands

## Past

In recent years, recommendations in guidelines for cutaneous melanoma have changed considerably. Although guideline recommendations for its indication differ slightly per country, it is believed that sentinel lymph node biopsy (SLNB) should be considered for patients with more than 1 mm Breslow thickness.^1^–^3^ Until recently, SLNB was performed for extra prognostic information to inform patients as optimally as possible about their prognosis. This study aimed to evaluate the trend in SLNB enactment during a 15-year period in The Netherlands.

## Present

Only 9761 (39.7%) of all eligible patients underwent SLNB. Although the trend showed an increase in SLNB enactment, from 39.1% in 2003, still only 47.8% of all eligible patients in 2014 underwent SLNB. Variables significantly associated with non-enactment were female gender, older age, and melanoma located on the head and neck.^4^

## Future

A positive SLNB has become the gateway to adjuvant immunotherapy for melanoma patients rather than to lymph node dissection.^5^ Because this is a major change in the reason for performing an SLNB, the authors believe their data underscore the fact that many eligible patients still are denied an SLNB, and much work remains to be done to ensure that this procedure will be performed when indicated. The authors hope their report contributes to more awareness with regard to SLNB and eventually to a better prognosis for melanoma patients.
